# Patient experiences and contextual appropriateness of universal screening for depression and suicide risk in HIV care: a qualitative study in Tanzania

**DOI:** 10.3389/frhs.2025.1557348

**Published:** 2025-04-24

**Authors:** Kim Madundo, Mirlene Perry, Judith Mwobobia, Ismail Shekibula, Elizabeth F. Msoka, Clotilda S. Tarimo, Victor Katiti, Blandina T. Mmbaga, David B. Goldston, Michael V. Relf, Brandon A. Knettel

**Affiliations:** ^1^ Department of Psychiatry and Mental Health, Kilimanjaro Christian Medical Centre, Moshi, Tanzania; ^2^ Kilimanjaro Christian Medical University College, Moshi, Tanzania; ^3^ Duke University School of Nursing, Durham, NC, United States; ^4^ Brown School, Washington University in St. Louis, St. Louis, MO, United States; ^5^ Duke Global Health Institute, Durham, NC, United States; ^6^ Kilimanjaro Clinical Research Institute, Moshi, Tanzania; ^7^ Department of Psychiatry & Behavioral Sciences, Duke University, Durham, NC, United States; ^8^ Duke Center for Global Mental Health, Duke University, Durham, NC, United States

**Keywords:** depression, suicide, screening, HIV, mental health, stigma, Tanzania, safety planning

## Abstract

**Introduction:**

Depression and suicidal thoughts and behaviour are remarkably common among people living with HIV worldwide, leading to a higher burden of disease, poor HIV care engagement, and death. Suicidal behaviour is criminalized in 20 countries worldwide, including Tanzania, where context-appropriate interventions are lacking.

**Methods:**

We describe the experiences of patients who were screened for depression and suicidal ideation by HIV clinic nurses, and how the socio-cultural context influences these experiences. This screening was the initial procedure in a randomized controlled clinical trial and parent study aimed at reducing suicide and depression and improving HIV care engagement in Kilimanjaro, Tanzania. We conducted in-depth interviews (IDIs) with 20 people living with HIV. Interviews were held 3 months post-enrollment of participants. Data was collected from July to November 2023. We referred to a brief screener developed for the trial, combining the PHQ-2 for depression and one question on suicidal ideation. IDIs focused on the experiences and appropriateness of being screened for depression and suicidal ideation, factors influencing these challenges, and opinions on the illegality of suicidal behaviour in Tanzania. Data was analyzed using Nvivo 14. Thematic analysis approach was utilized to identify, review and, label codes. Differences were resolved by the first three and final authors.

**Results:**

Our findings revealed high appreciation for the screening and occasional initial discomfort. Stigma, misinformation, and poverty contributed to mental health challenges. Patients favoured abolishment of laws against suicide due to their hindering support-seeking and impracticality for individuals in mental health crises.

**Discussion:**

In a mental health resource-limited setting, these findings highlight the need for targeted and integrated non-specialist screenings and interventions and outsourcing HIV and mental health support beyond healthcare facilities. Further research is needed to assess the sustainability of screening.

## Introduction

Tanzania has the sixth largest population of people living with HIV (PLHIV) in any country globally (1), at an estimated 1.7–1.9 million people (2). Depression and suicidality are common among PLHIV in Tanzania (2–4); depression is three times more prevalent among PLHIV than among the general population in Tanzania (5), and death by suicide occurs more than 100 times as often (6). The World Health Organization (WHO) estimates that over 3,000 Tanzanians die by suicide annually, and with rapidly increasing rates (2, 7). More than one in four of these deaths occur among PLHIV (8).

Mental health care resources in Tanzania are severely limited, with less than one mental health psychologist or psychiatrist per million people (9, 10). Additionally, routine screening and intervention for prevalent mental health challenges such as depression and suicidality are lacking in most healthcare settings, including HIV care (2, 4). These challenges underscore the urgent need for economical and enduring mental health solutions, such as integrating and task-shifting mental health services (4, 11), which can enhance the quality of life and have lifesaving benefits for both HIV care and suicide prevention.

Hindrances to effectively addressing mental health challenges in this setting include stigma, low awareness, and the minimal integration of mental health services into the overall healthcare system (2, 9, 12, 13). Additionally, Tanzania is among 20 countries globally where suicide is criminalized (14); Section 217 of the Penal Code states: “Any person who attempts to kill himself is guilty of an offence”. The criminalization of suicide likely perpetuates stigma and negatively affects patients' willingness to seek help for suicidal thoughts, resulting in numerous undetected and untreated cases (15). Recent literature from this setting reveals HIV and mental health care providers report this ruling as misguided and unethical (16).

In Tanzania, extreme levels of stigma towards both mental health and HIV exist; individuals underreport psychosocial challenges in the general medical system, have low self-efficacy in expressing these challenges, and are more inclined to utilizing traditional healers for psychosocial support (11, 17, 18). Additionally, socio-cultural factors such as the public opinions of mental health challenges play a key role in the experience of mental health care (19, 20). Considering these challenges, studies conducted in Kilimanjaro have emphasized the importance of establishing feasibility and cultural appropriateness of task-shifting brief nurse-led screening of depression and suicidality paired with counselling (9, 21). However, little is known about the patient experiences of these endeavours, and how living with mental health challenges in Tanzania may affect patient's experiences of receiving HIV care.

The IDEAS for Hope parent study in Kilimanjaro has implemented a brief screener for depression and suicidality during routine HIV care visits, prior to enrollment in a brief telehealth-based counseling intervention to reduce depression and suicidality and enhance HIV care engagement (2). This current study aimed to explore patients' experiences of this novel screening program, the reality of living with HIV, and how it relates to experiences of depression and suicidal thinking and/or behaviour in Tanzania.

## Materials and methods

### Study setting and design

The IDEAS for Hope randomized clinical trial was registered at clinicaltrials.gov (ID: NCT04696861). This trial aimed to assess the feasibility, acceptability, and efficacy of a newly culturally adapted three-session telehealth-based counselling intervention designed to address suicidality and depression among PLHIV in the Kilimanjaro region of Tanzania.

The initial step was universal screening for depression (Patient Health Questionnaire 2-tem version, PHQ-2) and a single item assessing suicidal ideation in the past month. Patients who screened positive for depression and/or suicidal ideation were invited to participate in the parent study. The current study explores patient's experiences of this novel screening procedure.

Enrolled participants randomized to the intervention arm received the telehealth counselling intervention, delivered via WhatsApp video call on a study-owned tablet located at one of four study sites, connected to a telehealth counsellor at Kilimanjaro Christian Medical Centre (KCMC). The intervention content covered in the three counselling sessions was informed by a four-pillar framework to address the target challenges; living a healthy life with HIV, being free of stigma, improving emotional stability, and meeting basic needs.

The screening and telehealth intervention were implemented at four HIV clinics in Kilimanjaro (9). The four study sites are all located in an urban setting in the Kilimanjaro region of Tanzania. KCMC is a zonal referral-level general hospital with a catchment area of approximately 15 million people. KCMC receives patient referrals from the other three sites for a variety of cases, including HIV care, often addressing the most complex health challenges. KCMC has six psychiatrists, however, provides outpatient mental health services only. Mawenzi Regional Referral Hospital (MRRH) is the designated tertiary-level facility for the Kilimanjaro region, receiving patient referrals from all six districts in the region, and is home to the only inpatient psychiatric unit in the Kilimanjaro region. There are no mental health specialists at Mawenzi RRH. Majengo Health Centre and Pasua Health Centre are smaller primary health care facilities that also provide HIV care. Majengo has one mental health professional and a Bachelor's-level counsellor, and Pasua has none and relies on support provided by a social worker and external referrals for mental health treatment.

All four facilities operate outpatient clinics for HIV care, which includes HIV testing, daily oral antiretroviral medication, medication monitoring, and HIV education offered free of charge or for a small administrative fee. At the time of this study, newly diagnosed patients attended biweekly or monthly outpatient HIV appointments and then, once they were established in care, transitioned to twice-yearly appointments.

### Screening procedure and assessment tools

HIV clinic nurses informed patients of the new mental health screening procedures upon their arrival for routine HIV clinic visits. Before individual screening, nurses provided brief group education sessions in the waiting room, intended to normalize mental health challenges and highlight their impact on quality of life.

Patients were screened in a private room by a nurse before their regular consultation with a doctor. The PHQ-2 assesses how often, over the past 2 weeks, the patient has had “little interest or pleasure in doing things” and has been “feeling down, depressed, or hopeless” (22). Items are responded to on a Likert-type scale from “0-Not at all” to “3-Nearly every day” for a total score of 0–6. This scale has been shown to have similar validity to the longer 9-item version of the PHQ while taking less time to administer (23). For this study, we used the standard cutoff score of three or higher to indicate likely depression.Nurses screened for suicidal thinking using one yes/no item derived from the Columbia Suicide Symptom Rating Scale (C-SSRS), “In the last month, have you had actual thoughts of killing yourself?” (24).

### Participant recruitment

The recruitment period for the parent IDEAS for Hope study ran from 5th March to 1st August 2023. The current study enrolment period lasted from 3rd July to 3rd November 2023. Patients were contacted 3 months post-screening, provided with information about the current study and invited to participate.

### Study procedure

We conducted qualitative in-depth interviews (IDIs) with 20 patients who were screened; 10 who screened positive for likely depression and suicidal thinking and were referred for additional counselling; five patients who screened positive for likely depression only (referred for a Problem Management + session); and five who screened negative for both depression and suicidal thinking (controls). IDIs on the current study were conducted 3 months post-enrolment, in Swahili in a private study office at KCMC by KM, a psychiatrist. Participants received reimbursement of 10,000 Tanzanian shillings (approximately $4.50 U.S.) for their time and transport costs.

Interviews lasted between 15 and 30 min; they began with collecting brief demographic information. IDIs focused on the respondents' experiences and actions during the mental health screening and referral process, perceived public opinions of PLHIV, depression and suicide, and factors contributing to these challenges among PLHIV. Other questions explored views on the health education provided to PLHIV and the criminalization of suicide in Tanzania.

### Qualitative data analysis

IDIs were audio recorded. Audio files were transcribed and translated into English by two bilingual research staff with Bachelor's degrees, a minimum of 2 years' research experience, and written and verbal fluency in both English and Swahili. The transcripts were then transferred to NVivo 14 for analysis. The study team used applied thematic analysis; to gain familiarity with the data, KM, JM, MP, and BAK first read through the transcripts independently and highlighted meaningful segments of the raw transcripts. They used an inductive process to identify, review, qualify, and label emergent themes and sub-themes. These themes were referred to as the co-authors read through the transcripts again and used to find codes, and thereafter prepare a report (25). The first three authors conducted initial coding, with BAK as the data auditor. KM, JM, MP, and BAK revised the themes, focusing on resolving differences. The inter-coder agreement was assessed by KM, JM, and MP on 30% of the transcripts being independently coded by a second coder and compared with the first. The mean inter-coder agreement was 95.2%, exceeding a pre-established threshold of 80%. Differences were resolved by the three authors and reflected in the final coding.

### Ethical approval and consent to participate

Clearance to conduct this study was granted by research committees at Kilimanjaro Christian Medical Centre (Protocol 1307, Certificate No. 2523), Duke University Health System (Protocol: Pro00107424-CR-3.2), and the Tanzanian National Institute for Medical Research (Protocol: NIMR/HQ/R.8c/Vol.I/2420). Before IDIs, all participants provided written informed consent.

## Results

We report the findings of IDIs with 20 participants, exploring (i) patients' experiences of a novel integrated and brief mental health screening in HIV care, (ii) contextual factors regarding living with mental health challenges in Tanzania and how these may affect patients' screening experiences and (iii) implementation gaps and opportunities to address these challenges among PLHIV.

The majority of participants (65%) were female. All of the respondents were black, with ages ranging between 22 and 75 years, and duration in HIV care ranging from 3 months to 28 years. See Table 1 for an overview of participant characteristics.

**Table 1:** Patient characteristics.

Participant ID	Age	Gender	Time in HIV care (years)
PT01	22	Female	15
PT02	62	Female	9
PT03	57	Male	17
PT04	38	Female	1
PT05	47	Female	3 months
PT06	49	Female	17
PT07	47	Female	15
PT08	58	Female	20
PT09	45	Male	5
PT10	48	Male	3
PT11	47	Female	3 months
PT12	48	Male	26
PT13	55	Female	13
PT14	46	Male	1
PT15	59	Male	20
PT16	28	Female	28
PT17	75	Female	16
PT18	65	Female	6
PT19	48	Female	4 months
PT20	45	Male	8

### Patients' experience of the screening process

The majority of the participants reported a positive experience with screening (*n* = 18). Participants respected the healthcare workers' position in asking the questions and felt that the screening presented a unique opportunity to discuss emotions and life challenges, something many had not done before. They felt relief and a sense of being supported and heard, while other participants summarized their screening experience as normal. One participant remarked:

“I felt very consoled because I felt as if I had found people to support me.”

PT 04

Age 38, female, screened positive for depression and suicidal thinking

Participants reported feeling seen and understood by the screening staff, and as a reminder to remain resilient:

“I assumed that this is a nurse and they have found a way to ask me this and a way to save me”

PT 02

Age 62, female, screened positive for depression and suicidal thinking

Other participants reported that the screening offered a moment for pause and introspection. The participants further emphasized that the screening questions felt brief yet all important, that none of the questions was unnecessary or harmful, and that it did not add burden to their usual appointment time.

However, two participants reported a negative experience with screening; these occasions were both short-lived. One described a feeling of worry:

“I was a bit worried because they are not the easiest questions to be asked.”

PT 14

Age 46, male, screened negative

The second participant expressed a feeling of transient sadness after being reminded about their difficult past.

“I felt sad while being screened. But I am grateful to be here and very relieved.”

PT 02

Age 62, female, screened positive for depression and suicidal thinking

**Figure 1:**
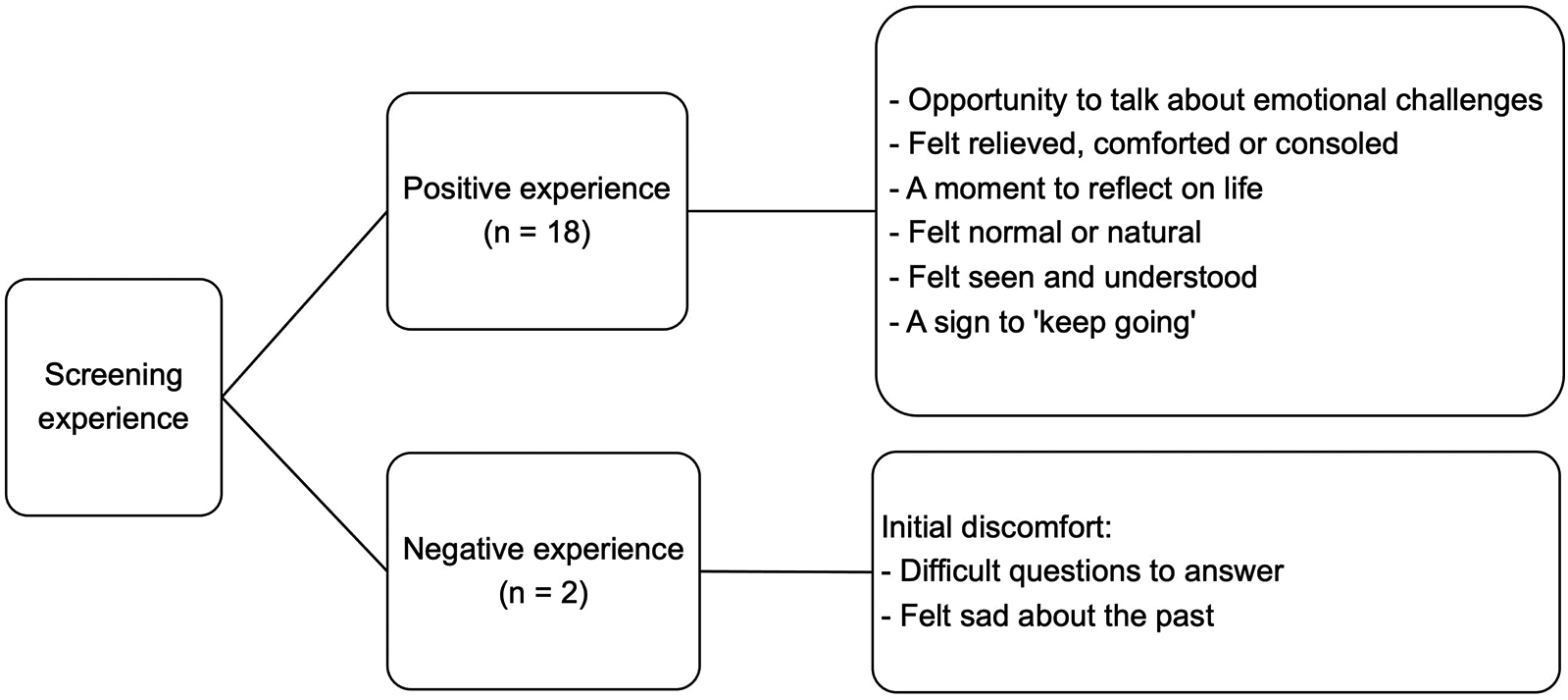
Experiences with screening for depression and suicidal thinking.

This duality highlights the complexity of discussing mental health, in this socio-cultural context, and particularly while in a crisis. Additionally, the relief post-screening indicated that the screening process served not only as a means of assessment but also as a therapeutic encounter, providing participants with a rare opportunity to express their feelings. See Figure 1 for an overview of these findings.

### Contextual factors regarding HIV

Participants reported a range of factors that predisposed to, perpetuated or protected them from mental health challenges while living with HIV.

### Society's views on HIV

Participants shared a complex view on societal attitudes towards PLHIV, noting both support and considerable stigma. Participants reported that while some people recognize HIV as a manageable chronic disease and encourage early treatment, others described unsupportive and stigmatizing behaviors.

“Society knows that when you are infected, you should hurry to the hospital and start receiving treatment, and you can continue with your life normally.”

PT 12

Age 48, male, screened negative

“People with a positive attitude are very few. Most people don't have enough knowledge about it and do not care about it since they are not the ones who are sick.”

PT 10

Age 48, male, screened positive for depression

Participants highlighted common misconceptions and ignorance, such as the belief that casual interactions could lead to infection, contributing to stigma, as evidenced by these quotes:

“…you also ask yourself why people are treating you the way they are treating you while it has been announced on the media that talking to someone living with HIV or if you share food with them, it cannot spread the disease.”

PT 10

Some participants reported that societal values intensify stigma against PLHIV, associating their condition with promiscuity or personal failure and that this stigma can lead to isolation, even from family members and health providers, worsening emotional distress.

“People tell you that it is because of your stupidity that you got sick, and they say you were not settled with one partner, that is why you got sick.”

PT 10

Age 48, male, screened positive for depression

A participant noted that there was a need for governmental protection against discrimination, advocating for policies that could decrease the marginalization of PLHIV:

“In some places, once they know that you are infected, they stigmatize you, and they see you as if you are already dead. Even relatives isolate themselves from you. That can also cause one to be depressed and suicidal, and that is the reason why we were asking the government to have more protection for people living with HIV.”

PT 14

Age 46, male, screened negative

### Risks for depression and suicide among PLHIV

Most participants highlighted isolation (*n* = 10) and stigma (*n* = 9) as significant risks for depression and suicide among PLHIV. Self-stigma often led to isolation due to the fear of other people in the community finding out about their HIV diagnosis.

Participants reported that isolation from family, often underscored by HIV stigma, caused anxiety for PLHIV. This situation resulted in individuals either concealing their struggles or experiencing diminished self-esteem.

“People around you just look at you weirdly. They speak ill of you, and you almost always know that they are speaking about you. You feel so unimportant… So, things like that cause you to be very depressed, you have trouble sleeping at night, and you get to think a lot. Some people are unable to bear the pains of this and end up killing themselves.”

PT 10

Age 48, male, screened positive for depression

Participants also reported poverty (*n* = 8) as a risk for mental health challenges. Not having a stable income triggered feelings of inadequacy. Additionally, food insecurity often led patients down a path of depression and suicidal ideation.

“You have no specific job, or your business is not profitable—you will obviously sink into depression and suicide.”

PT 07

Age 47, female, screened positive for depression

One participant reported a fear of dying from AIDS as a risk for depression and suicide. The belief that an HIV diagnosis was a death sentence caused fear and reluctance to disclose the diagnosis and contributed to depression and suicidal ideation.

“I did not tell my family anything about my sickness, and even up to today, they do not know the truth. I was very depressed about the diagnosis. I would think of the people who died from AIDS and the way they were so thin and looked like skeletons, and I was afraid of being like that.”

PT 15

Age 59, male, screened negative

### Contextual factors regarding suicide

#### Society's views on suicide

Most participants viewed society as either condescending (*n* = 13) or indifferent (*n* = 5) towards people expressing suicidal thoughts or behaviour.

“There are those who say, ‘she knew she was going to die that is why she has decided to finish herself off early’”.

PT 11

Age 47, female, screened positive for depression

“The one who doesn't care if you live… They see that you are dirt, you have no benefit in the family.”

PT 02

Age 62, female, screened positive for depression and suicidal thinking

Participants highlighted ignorance towards suicide and other mental health challenges, and that it was less common for society members to be curious about what led to a mental health crisis (*n* = 4) or being supportive (*n* = 2).

“There are some who ask themselves why someone did that… and why did they not ask for advice before.”

PT 10

Age 48, male, screened positive for depression

“The ones who keep you safe, they won't let you leave. They want you to keep on [living] peacefully.”

PT 02

Age 62, female, screened positive for depression and suicidal thinking

#### The illegality of suicidal behaviour in Tanzania

Participants were asked about their opinion of the illegality of suicidal behaviour in Tanzania. The majority, 17 participants, were critical of this law. Among them, 15 participants further commented that the law against suicide had no impact on suicidal behaviour, which rendered it useless:

Four participants commented that creating awareness and educating the public on mental health challenges would be more impactful than criminalizing suicide.

“If they get a good counsellor, they may change their minds.”

PT 16

Age 28, female, screened negative

Seven participants remarked that this penal code worsens the stigma against mental health challenges, hinders help-seeking, and isolates people who are struggling when they need help most.

“Because of the law, you may tell a relative that you have a problem, and instead of them helping you, they stigmatize you even more.”

PT 11

Age 47, female, screened positive for depression

Three participants were in favor of suicidal behavior being considered a crime, stating that it would instil fear in one's mind and prevent a suicide attempt.

### Implementation gaps and opportunities

The participants identified gaps in current care, and provided a range of recommendations to improve the care and support of individuals living with HIV, including those dealing with depression or suicidal ideation.

All participants directed at least one recommendation to healthcare professionals in increasing access to and acceptability of mental health screenings: to do more community outreach and screening, educate society on mental health challenges and HIV, and improve their counselling skills.

“And they should not only talk to people who are infected but also to people who are not infected, educating them on how to live with PLHIV.”

PT 10

Age 48, male, screened positive for depression

“We can have people move from house to house offering education to people or from street to street giving flyers.”

PT 19

Age 48, female, screened negative

Participants (*n* = 16) emphasized that HIV clinics largely remained the only source of information on living healthily with HIV. This highlights current gaps in awareness efforts, for both PLHIV and uninfected individuals.

Interviewer: “Besides the hospital, is there any other place where you get your information on how to safely live with HIV?”

Respondent: “No*.*”

PT 11

Age 47, female, screened positive for depression

“The only information we receive is from the hospital.”

PT 03

Age 57, male, screened positive for depression and suicidal thinking

Some participants (*n* = 6) suggested involving families and caregivers of PLHIV in screening programs. According to some participants, this would help reduce HIV stigma and create a more knowledgeable and supportive environment for those affected.

“Relatives [need to be screened] too. And counselled when the relative discloses their status. Because if you don't tell them, that is where the stigmatization begins.”

PT 07

Age 47, female, screened positive for depression

The government was called upon (*n* = 5) to enforce protections for PLHIV. Participants noted that stigma and discrimination, and by consequence, mental health challenges, were a result of weaknesses in these protections.

“The government has to come in…they need to emphasize these laws so that [people with HIV] are not discriminated upon or hurt.”

PT 10

Age 48, male, screened positive for depression

## Discussion

Our findings offer insights into the experiences of PLHIV undergoing mental health screenings, informed by relevant contextual factors that may influence these experiences: Depression and suicidal thoughts and behaviour are common among PLHIV (3, 4, 26). Suicide rates are increasing in Tanzania (7), while suicidal behaviour is criminalized (27). Resources to address these mental health challenges within HIV care settings are urgently lacking (9, 10). One potential solution is to develop interventions that integrate mental health services in non-mental health settings to improve mental health treatment capacity (9).

### Implications on clinical practice

The integration of mental health screening in HIV care enhances the early detection of mental health challenges, brief intervention, and referral to a higher level of care as needed (9). The screening process led to overall positive feelings and experiences for the majority of participants, which contributed to improved engagement in HIV care. These findings underscore the appropriateness and significance of the integration of mental health screening into this resource-limited socio-cultural context. The results further demonstrate the abilities of non-mental health care providers in these settings to astutely identify and support patients in need (9). Previous literature has similarly encouraged the integration of mental health screening into non-mental health settings and spotlighted the underutilisation of this practice (28–30).

The participant quotes offer a reminder of the paramount need for individualized HIV education; to patients, regardless of their duration engaged in HIV care, but also to treatment supporters. Stigma towards both HIV and mental health, and subsequent relational tension can be reduced by addressing misconceptions timely (31, 32).

### Implications on policy

Integrating mental health screening into existing care frameworks enabled patients to access vital opportunities for mental health screening and referrals where necessary, addressing significant gaps in patient care. For the entire sample in this current study, this was the primary exposure to mental health services in any form. Our findings offer a necessary and timely reminder to policymakers that innovations are necessary to improve comprehensive care packages, and that effective interventions need not be resource intensive. Global literature has emphasized the need for enhanced government and policy support and health reform (33, 34), further stating that brief mental health interventions integrated into the health system can reduce premature rates of morbidity and mortality (30).

Participants overwhelmingly reported experiencing discrimination due to the criminalization of suicide in Tanzania. The current legal framework poses a significant barrier to the welfare of people experiencing severe mental health challenges and spotlights the need for the government to urgently reconsider such biased policies. The criminalization of suicide—a common experience for PLHIV—is in contradiction with Tanzania's HIV and AIDS Act of 2008 which prohibits regulations that directly, or by implication, discriminates against PLHIV, and requires the highest standard of physical and mental health for PLHIV. Reconsidering laws related to suicidal behavior in a socio-cultural context such as Tanzania could mitigate HIV and mental health stigma for PLHIV, thereby enhancing care-seeking behavior and access to care. Literature exploring this transition towards decriminalization of suicidal behavior has identified positive correlations between reframing suicide as a mental health challenge rather than a crime (35). Additionally, the World Health Organization published a policy brief with case samples of countries that recently decriminalized attempted suicides, along with the positive impact of this transformation (36).

### Implications for future research, strengths, and limitations

Education remains crucial for living healthily with HIV, emphasizing the need for ongoing and targeted education for PLHIV and the broader community. This should include outlets such as radio, television, and social media. Studies from various cultural settings have demonstrated the effectiveness of HIV education campaigns and recommended areas of focus when establishing HIV education campaigns (37, 38).

Future research could delve into the sustainability of mental health screening in HIV care settings and determine how community-centered educational campaigns can impact HIV and mental health stigma (39–41). Similar to our findings on lacking sources of HIV information beyond healthcare settings, recent literature reveals a significant gap also exists in communities (42, 43). An apparent opportunity, therefore, is for community-based organizations and community-engaged research to evaluate and refine existing educational campaigns with both HIV and mental health components (44).

The research highlights the importance of mental health screening in settings with limited mental health resources. To the best of our knowledge, our study is the only one from Tanzania to report the experiences of patients being screened for depression and suicidal ideation in a non-mental health setting. Recognizing the increasing burden of mental health challenges and the ongoing lack of resources to address them, it is paramount to understand the experiences of patients to support the improvement of services, informed by comprehension of the contextual background.

One of the study's limitations is its focus on a population from one urban area in Tanzania with a unique socio-cultural setting. This could affect the reproducibility of the findings in other areas.

## Conclusion

Patients in HIV care who were screened for depression and suicidal thinking found the screening process to be beneficial. Many experienced stigma, interpersonal conflict, and reduced engagement in HIV care. Participants reported improved awareness of mental health and motivation to continue engaging in HIV care. The majority of participants opposed the criminalization of suicide.

The study underscores the substantial impact of mental health challenges on the lives of PLHIV, including the heightened suicide risk. We emphasize the urgent need for early identification and intervention for these challenges, increasing HIV and mental health education, and supporting the integration of mental health services beyond traditional mental health settings.

## Data Availability

De-identified data supporting the conclusions of this article will be made available by the authors, without undue reservation.
